# First insight of genetic diversity, phylogeographic relationships, and population structure of marine sponge *Chondrosia reniformis* from the eastern and western Mediterranean coasts of Tunisia

**DOI:** 10.1002/ece3.8494

**Published:** 2022-01-11

**Authors:** Maha Moussa, Sarra Choulak, Soumaya Rhouma‐Chatti, Noureddine Chatti, Khaled Said

**Affiliations:** ^1^ Laboratory of Genetics, Biodiversity, and Bioresources Valorization (LR11ES41) Higher Institute of Biotechnology of Monastir University of Monastir Monastir Tunisia

**Keywords:** *Chondrosia reniformis*, COI mtDNA, phylogeography, Siculo‐Tunisian Strait, Tunisia coasts

## Abstract

Despite the strategic localization of Tunisia in the Mediterranean Sea, no phylogeographic study on sponges has been investigated along its shores. The demosponge *Chondrosia reniformis*, descript only morphologically along Tunisian coasts, was chosen to estimate the influence of natural oceanographic and biogeographic barriers on its genetic differentiation and its Phylogeography. The cytochrome oxidase subunit I (COI) gene was amplified and analyzed for 70 Mediterranean *Chondrosia reniformis*, collected from eight localities in Tunisia. Polymorphism results revealed high values of haplotype diversity (*H*_d_) and very low nucleotide diversity (*π*). Thus, these results suggest that our sponge populations of *C*. *reniformis* may have undergone a bottleneck followed by rapid demographic expansion. This suggestion is strongly confirmed by the results of neutrality tests and “*mismatch distribution*.*”* The important number of haplotypes between localities and the high genetic differentiation (*F*_st_ ranged from 0.590 to 0.788) of the current *C*. *reniformis* populations could be maintained by the limited gene flow *N_m_* (0.10–0.18). Both haplotype Network and the biogeographic analysis showed a structured distribution according to the geographic origin. *C*. *reniformis* populations are subdivided into two major clades: Western and Eastern Mediterranean. This pattern seems to be associated with the well‐known discontinuous biogeographic area: the Siculo‐Tunisian Strait, which separates two water bodies circulating with different hydrological, physical, and chemical characteristics. The short dispersal of pelagic larvae of *C*. *reniformis* and the marine bio‐geographic barrier created high differentiation among populations. Additionally, it is noteworthy to mention that the “Mahres/Kerkennah” group diverged from Eastern groups in a single sub‐clade. This result was expected, the region Mahres/Kerkennah, presented a particular marine environment.

## Graphical abstract

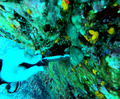

## 1 INTRODUCTION

*Phylum Porifera*, commonly known as sponges, are the evolutionary oldest multicellular animal. These invertebrates are found in all oceans and at all depths (Hooper & Van Soest, 2002; Van Soest et al., 2012). According to the World *Porifera* Database, the number of described taxa are more than 9000 (Van Soest et al., 2021). The latest classification divides sponges into four classes; *Calcarea*, *Hexactinellida*, *Homoscleromorpha*, *and Demospongiae*, to which more than 90% of sponge species belong. Unlike the other animals, sponges are the simplest group that lacks true tissues and organs. They are only formed by specialized cell types (e.g., choanocytes and pinacocytes), which are embedded in a complex matrix called mesohyl (Junqua et al., 1974; Simpson, 2012). Despite their simple morphology, sponges’ genome is complex (Harcet et al., 2010). Due to their higher filtering capacity, sponges play interesting roles in biogeochemical cycling and in the benthic‐pelagic coupling of nutrients within the ecosystem (De Goeij et al., 2013; Lesser, 2006; McMurray et al., 2017). Contrarily to most benthic organisms, these invertebrates have the capacity to pump large volumes of seawater through the water column. Crossing through the body of the sponge, seawater is chemically transformed due to feeding, excretion, and the activities of microbial symbionts, with significant effects on the carbon and nutrient cycling (Pawlik & McMurray, 2020). To defend against predators, and pathogens, sponges have developed numerous secondary metabolites, which present a high biotechnological potential in different domains (Genta‐Jouve & Thomas, 2012).

The sponge can reproduce sexually (gametes are produced from two types of somatic stem cells) or asexually. Sexual reproduction in sponges is varied, it can be gonochoric, sequential, or simultaneous hermaphrodites, it also can be through viviparity or oviparity (Maldonado, 2006). Their larvae are characterized by low dispersal potential (Vacelet, 1999). This disperse over short distances potential has important consequences for the connectivity and genetic structuring of sponge populations (Avise, 1994; Scheltema, 1971). Thus, larval dispersal potential is a key factor that can be used to understand the spatial patterns of genetic diversity, which is the main goal of phylogeographic studies (Cowen & Sponaugle, 2009; Palumbi, 2003). Phylogeography, which combines genetic and geographic data, allows comprehension of the distribution of genetic differentiation in terrestrial and aquatic ecosystems. Thus, this approach confers to understanding the spatial patterns of genetic diversity and both the historical and contemporary factors acting on taxa (Avise, 2000; Rissler, 2016). Moreover, phylogeographic studies are substantial for the development of effective conservation strategies in the increasingly threatened marine realm (Moritz, 2002; Moritz & Faith, 1998).

Despite its strategic localization, opening on the sides of the two Mediterranean sub‐basins separated by the Siculo‐Tunisian Strait, no phylogeographic study on sponges has been investigated along Tunisian shores. Thus, only morphological descriptive studies on sponges have been mentioned by Ben Mustapha et al. (2003), Ben Mustapha et al. (2007), Bouamama et al. (2009), and Zarrouk et al. (2005). Thereby, we chose the sponge *Chondrosia reniformis*, which is present along Tunisian shores according to the work of Bouamama et al. (2009) to investigate its phylogeographic structure and to estimate the influence of natural oceanographic and biogeographic barriers on its distribution.

*Chondrosia reniformis* Nardo, 1847 (*Demospongiae*, *Chondrosiidae*), is a thick encrusting, smooth, and cushion‐shaped sponge. Under the effect of light intensity, its body colors vary to white, brown, black, and sometimes orange. This species was considered to have a worldwide distribution, including the Atlantic, Pacific, and Indian Oceans and Mediterranean Sea (Di Camillo et al., 2011; Idan et al., 2020; Lazoski et al., 2001). *Chondrosia reniformis* lives on shady rocky coasts at a depth of up to 50 m and it can be found in shallow, mesophotic, and oligotrophic habitats (Di Camillo et al., 2011; Idan et al., 2020).

This demosponge is a gonochoric broadcaster sponge that also can reproduce asexually via drop‐like propagules (Di Camillo et al., 2011; Riesgo & Maldonado, 2008). Both the dispersal capability of the lecithotrophic larvae and the gamete's dispersal are probably low. Its reproductive cycle is believed to be influenced by temperature (Idan et al., 2020). Among areas, oogenesis appears to be varied from seasonal to continuous, it is obtained before the temperature peak around May to August (Di Camillo et al., 2011; Riesgo & Maldonado, 2008). Spermatogenesis in *C*. *reniformis* seems to be rapid and probably synchronized with the last developmental stage of the oocytes (Di Camillo et al., 2011).

The incorporation of the foreign particles to strengthen its body, the creeping phenomenon used to reproduce asexually; the reproductive cycle and the high production of collagen have been extensively studied for *Chondrosia reniformis* (Bavestrello et al., 1995; Bonasoro et al., 2001; Di Camillo et al., 2011; Fassini et al., 2014, 2017; Nicklas et al., 2009; Pozzolini et al., 2018; Silva et al., 2016). However, few genetic studies have been performed on this species. Indeed, Lazoski et al. (2001) have investigated the levels of genetic variation within and between geographically distant populations of this species from the Atlantic (North and South America) and Western Mediterranean sea coasts.

In the last two decades, DNA sequences have been extensively used to understand the evolutionary history and spatio‐temporal genetic divergence of species, and it is mitochondrial DNA that is commonly used. Indeed, since its maternal inheritance without recombination, high mutational rate, shorter coalescence times, and high copy numbers in the organism (Avise, 2000, 2009; Palumbi et al., 2001), this genome is commonly used as a genetic marker to identify the taxa as well as to investigate phylogeographic relationships in most marine organisms (Avise, 2000). However, no nuclear or mitochondrial DNA molecular studies have been undertaken on *C*. *reniformis* to analyze its population structuring and its phylogeography. The only studies carried out have focused on the cytochrome oxidase subunit I DNA marker (COI) in order to position the genus *Chondrosia* in the phylogenetic tree of demosponges (Riesgo et al., 2014; Rot et al., 2006; Rua et al., 2011; Vacelet et al., 2000; Villamor et al., 2014; Xavier et al., 2010).

**FIGURE 1:**
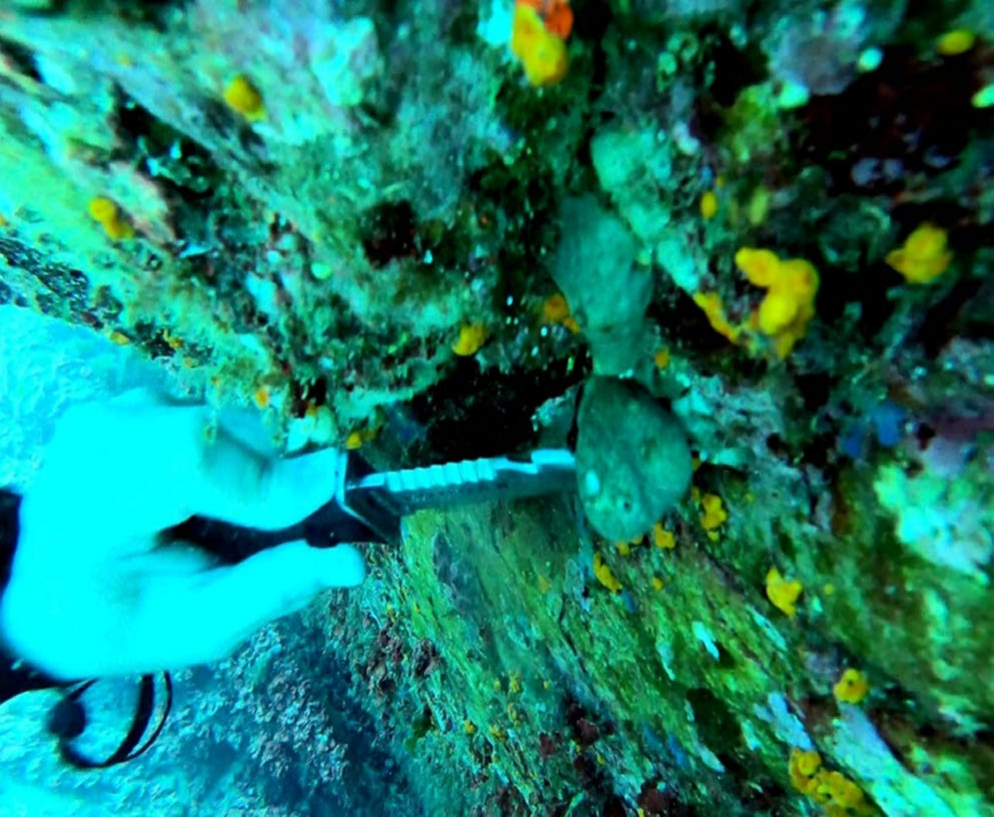
Photo of *Chondrosia reniformis* specimen collected by Wissem Dallai diver from the Beja locality in September 2020 at 10 depth

The aim of this study, using COI mitochondrial DNA marker is to estimate levels of diversity and differentiation of Tunisian coastal populations of the two east and west Mediterranean basins, to analyze the effects of natural oceanographic and biogeographic barriers between these two basins and finally to establish for the first time the phylogeography of *Chondrosia reniformis* along its Tunisian coastal distribution (Figure 1).

## 2 MATERIAL AND METHODS

### 2.1 Sample collection and genomic DNA extraction

**FIGURE 2:**
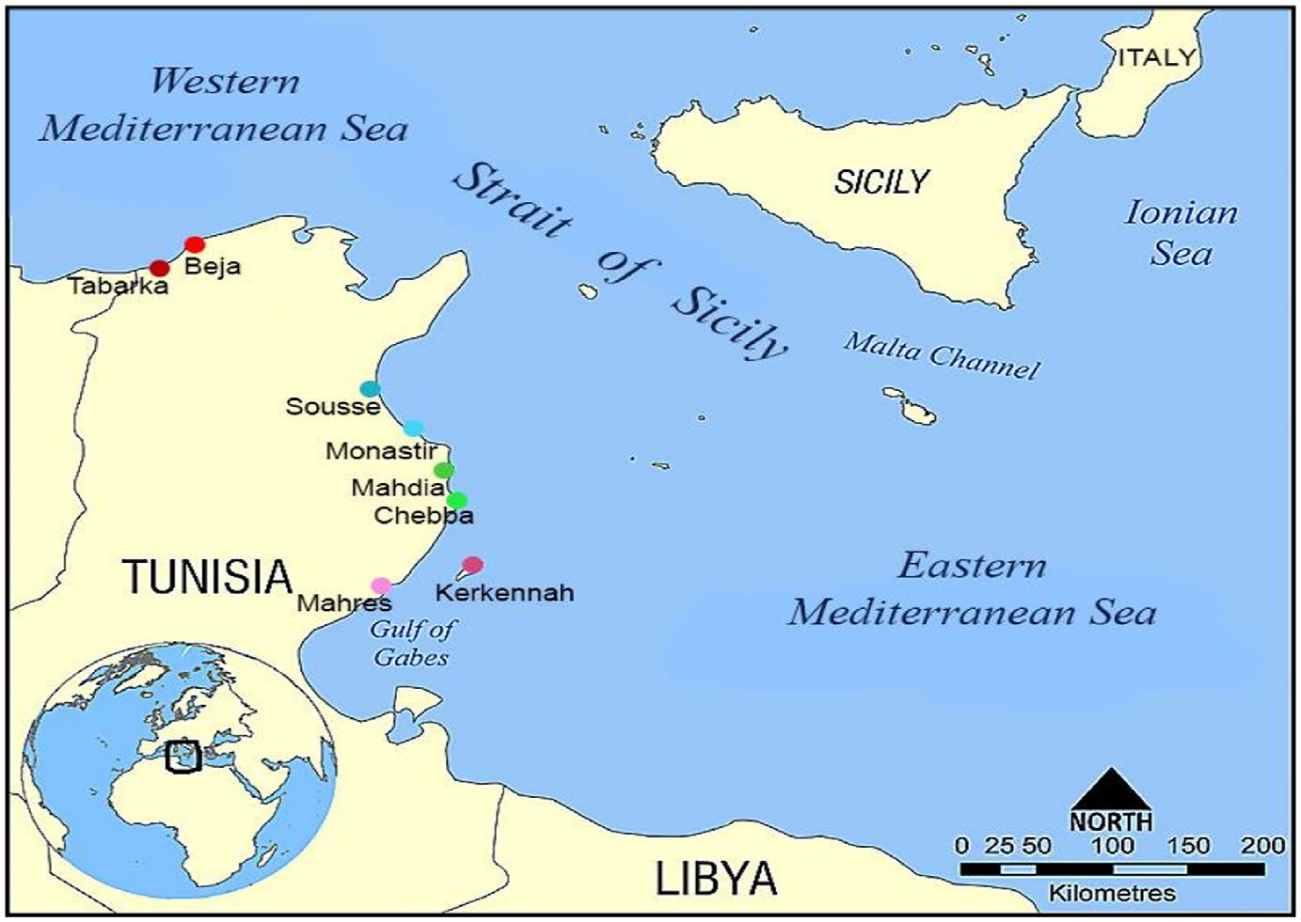
Geographic distribution of *Chondrosia reniformis* samples

A total of 70 specimens of *Chondrosia reniformis* (Nardo, 1847) were collected, between January and September 2020, from eight sampling locations along the Tunisian coasts (Figure 2, Table 1). These samples covered the western and eastern board of the Mediterranean (Tabarka, Beja, Sousse, Monastir, Mahdia, Chebba, Mahres, and Kerkennah). From each specimen, 100 mg of tissue were preserved in 100% ethanol and stored at 20 °C for subsequent DNA extraction. DNA of sponge specimens was extracted using EZ‐10 Spin Column Kits (Bio BASIC INC, Canada) as described by the manufacturer. DNA quantity and quality were performed using a spectrophotometer (Gold S54T, Shanghai) and agarose gel electrophoresis (Sambrook et al., 1989).

**TABLE 1:** Information on demosponge *Chondrosia reniformis* sampling including collection region, collection site, number of specimens (*N*), and geographic coordinates

Collection region	Collection site	*N*	Geographic coordinates
Western Mediterranean	Beja	12	37° 06’ 14” N, 8° 58’ 51” E
	Tabarka	8	36° 57’18” N, 8° 45’ 18” E
Eastern Mediterranean	Monastir	18	35° 46’ 40” N, 10° 49’ 34” E
	Sousse	6	35° 49’ 34” N, 10° 38’ 24” E
	Mahdia	5	35° 30’ 16” N, 11° 03’ 43” E
	Chebba	12	35° 14’ 14’ N, 11° 6’ 54” E
	Mahres	4	34° 31’ 39” N, 10° 30’ 3” E
	Kerkennah	5	34° 39’ 29” N, 11° 04’ 07” E
	All datasets	70	

### 2.2 Mitochondrial DNA amplification and sequencing

The mitochondrial fragment of the cytochrome oxidase subunit I (COI) gene was amplified using a pair of universal primers (COI‐Frwd: 5'‐GGTCAACAAATCATAAAGAYATYGG‐3’; COI‐Rev: 55'‐TAAACTTCAGGGTGACCAAARAAYCA‐3’) (Folmer et al., 1994). PCR reactions were performed in a total volume of 25 μl including 2 μl (25 ng/μl) of DNA, 2.5 μl of PCR Buffer (10× final concentration), 3.2 μl of MgCl2 (20 mM), 0.5 μl of each primer (10 μM),0.5 µl of dNTP mix (10 mM), 0.1μl (1 U/μl) of Taq DNA polymerase, and sterile ddH2O. PCR amplifications were carried out in an Applied Biosystems^®^ 2720 Thermal Cycler, programmed to perform an initial denaturation at 94°C for 2 min; followed by 35 cycles at 94°C for 50 s, 52°C for 55 s, and 72°C for 1 min; and a final extension at 72°C for 7 min (Duran et al., 2004 with modifications). Amplicons were separated on 1.5% agarose gels at 100 V. The agarose gel was photographed by a Compact Digimage System, UVDI series (Major Sciences, USA).

Amplified PCR products were purified and sequenced (Sanger et al., 1977); sequences were aligned using ClustalW (Thompson et al., 1994) implemented in Bioedit (Hall, 1999).

### 2.3 Statistical analyses

Since the number of samples from each locality is unequal, we divided the sample into four groups based on geographic proximity (Beja/Tabarka, Monastir/Sousse, Mahdia/Chebba and Mahres/Kerkennah).

The level of DNA polymorphism, the haplotype diversity (*H*_d_; Nei, 1987) as well as the nucleotide diversity (*π*; Nei, 1987; Tajima, 1983), were measured for each group and for the total datasets using DnaSP version 5.10 (Librado & Rozas, 2009). The percentages of GC and AT, the number of variable and parsimony‐informative nucleotides sites were calculated with MEGA version 7.0.18 (Kumar et al., 2016).

The demographic history of the Mediterranean population of *C*. *reniformis* was investigated. The mismatch distribution test was performed with DnaSP v5.10.01 (Librado & Rozas, 2009) for all datasets and each group. To study the hypothesis of population expansion, additional tests were performed using the total number of mutations: Tajima's *D*‐test (Tajima, 1989), Fu's Fs test (Fu & Li, 1993), raggedness index (rg) and Ramos‐Onsins, and Rozas's R2 test (Ramos‐Onsins & Rozas, 2002). These analyses were executed using coalescent simulations implemented in DnaSP software, with 1000 simulated re‐sampling replicates.

### 2.4 Phylogeographic analysis and Genetic differentiation

To infer the relationships of *C*. *reniformis* haplotypes, we used the NETWORK software (Bandelt et al., 1999). Phylogenetic reconstructions were performed using (1) *Neighbor*‐*Joining* method in MEGA v6.06 (Tamura et al., 2013), (2) *Bayesian MCMC* (Markov Chain Monte Carlo method) analyses, in MrBayes v. 3.2.2 (Ronquist et al., 2012). Bayesian analysis was performed using the HKY + I + G model, as determined by the JModel Test (Posada, 2008) using the model correction based on AIC (Hasegawa et al., 1985). One sequence from *Chondrilla nucula* (GenBank accession numbers: EF519598.1) was used as outgroup.

The analysis of molecular variance (AMOVA) (Excoffier et al., 1992) was conducted by Arlequin 3.5 software (Excoffier & Lischer, 2010) to assess the level of genetic differentiation of Tunisian *Chondrosia reniformis* populations. Two supplementary AMOVA tests were carried out: for the first analysis, we tested the genetic variation between the four groups according to geographic proximity: Beja/Tabarka, Monastir/Sousse, Mahdia/Chebba and Mahres/Kerkennah. The second analysis was performed to evaluate a comparison between the group Mahres/Kerkennah and the other localities. In addition, the genetic differentiation for both eastern and western Mediterranean localities in Tunisia was tested too. All AMOVA analyses were calculated with 10,000 permutations under null distributions.

The extent of genetic differentiation between populations was estimated using the fixation index *F*_ST_ and the gene flow (*N_m_*) (Hudson et al., 1992). Values were calculated with 1000 data permutations using the software DnaSP v 5.10.01 (Librado & Rozas, 2009).

## 3 RESULTS

### 3.1 Genetic diversity and molecular evolution

A total of 70 *C*. *reniformis* COI sequences were obtained. Mitochondrial sequences varied from 619 to 727 pb. Among them, 30 different haplotypes were specified; 63 sites were variable and 51 were parsimony informative. Polymorphism analysis revealed high values of haplotypic diversity (*H*_d_) and very low nucleotide diversity (*π*). The percentage of GC is from 40% to 42%, and the AT percentage is from 58% to 60% (Table 2).

**TABLE 2:** Sampling information and diversity measures for the populations of *Chondrosia reniformis* studied

Collection site	N	Geographic group	*N*_h_	*H*_d_	*Π*
Beja Tabarka	12 8	Beja/Tabarka (20)	15	0.952	0,00817
Monastir Sousse	18 6	Monastir/Sousse (24)	7	0.707	0.0033
Mahdia Chebba	5 12	Mahdia/Chebba (17)	4	0.713	0.00418
Mahres Kerkennah	4 5	Mahres/Kerkennah (9)	4	0.833	0.00714
All datasets	70		30	0.939	0.00875

Abbreviations: *N*_h_, number of haplotypes; *H*_d_, haplotype diversity; *π*, nucleotide diversity.

**FIGURE 3:**
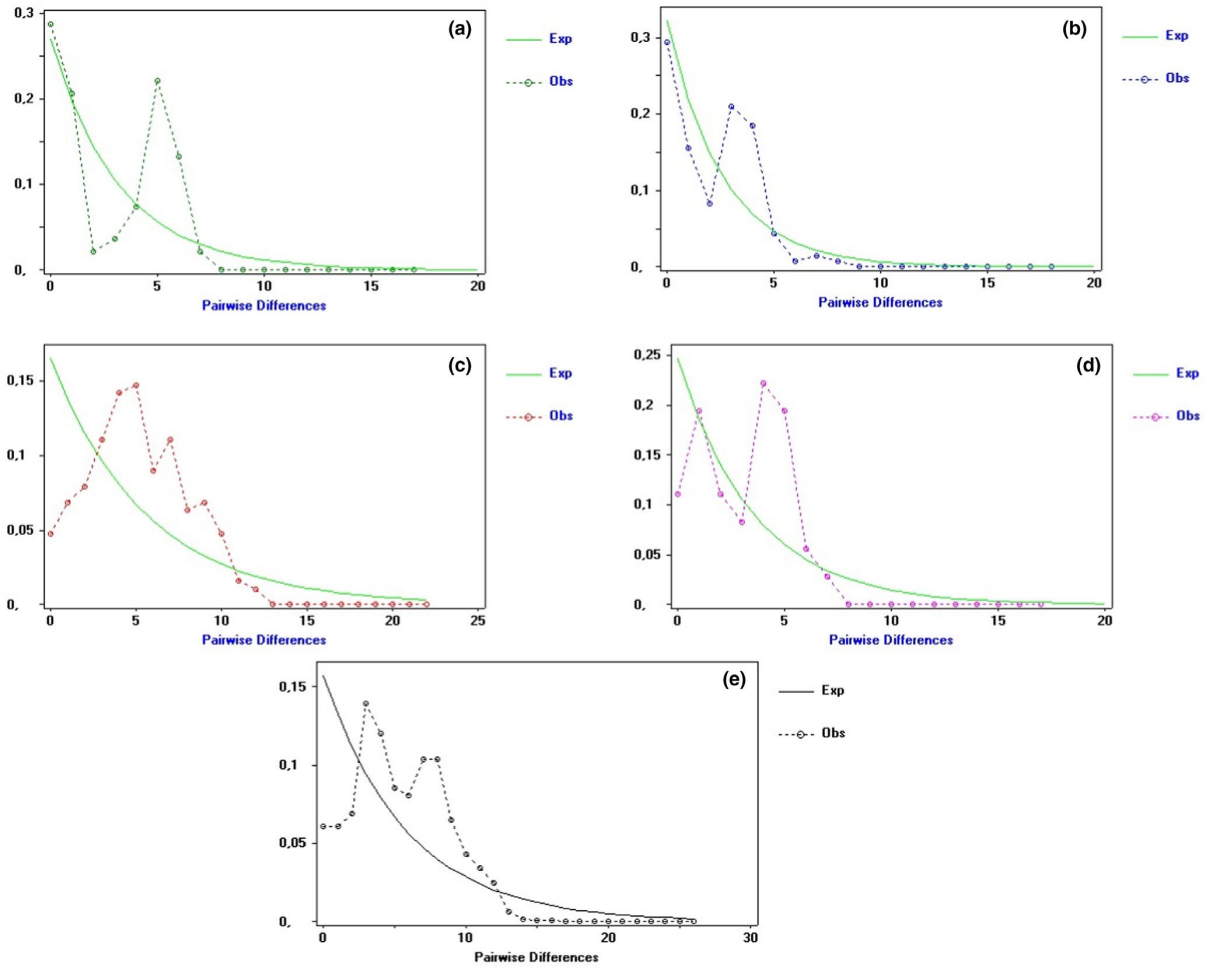
Pairwise mismatch distribution among groups; (a) Mahdia/Chebba, (b) Monastir/Sousse, (c) Beja/Tabarka, (d) Mahres/Kerkennah and (e) all datasets

Selective neutrality was estimated by Tajima (1989) and Fu and Li (1993) tests. These statistic tests were negative and insignificant for the three groups (Beja/Tabarka, Monastir/Sousse, and Mahdia/Chebba) and all datasets (Table 3). Selective neutrality tests for the group “Mahres/Kerkennah” were positives but insignificant. The overall (for the entire sample) negative values resulting from both tests indicate that there is an excess of rare mutations in the populations but the excess is statistically nonsignificant. Alternatively, these values can result from balancing selection on a nearby locus, although studies demonstrating direct or indirect selection (through hitchhiking) on the mitochondrial genome in natural populations are rare (Ruiz‐Pesini et al., 2004). Population size changes or “*mismatch distributions*” were tested for the four different groups and all datasets (Figure 3). Population size changes showed unimodal distribution, for all tested localities and all datasets, suggesting a case of populations in demographic expansion.

**TABLE 3:** Tajima's *D*, Fu's *F*S, Ramos‐Onsins and Rozas's *R*2 tests and raggedness index (*rg*) for each group of *Chondrosia reniformis* as well as for the entire sample

Geographic group	*D*	*FS*	*R*2	*Rg*
Beja/Tabarka	−0.83905	−6,489	0.160	0.092
Monastir/Sousse	−0.95817	−2,531	0.161	0.091
Mahdia/Chebba	−0.31203	−0,672	0.161	0.09
Mahres/Kerkennah	1.31944	1,071	0.163	0.089
All datasets	−1.14657	−12,487	0.096	0.053

We also calculated Ramos‐Onsins and Rozas's *R*2 and the raggedness index under the demographic expansion model for each population. We found that all populations had a nonsignificant raggedness index, which indicates that data has relatively good fit to a model of a population in demographic expansion (Harpending, 1994).

### 3.2 Phylogeography and genetic differentiation

**FIGURE 4:**
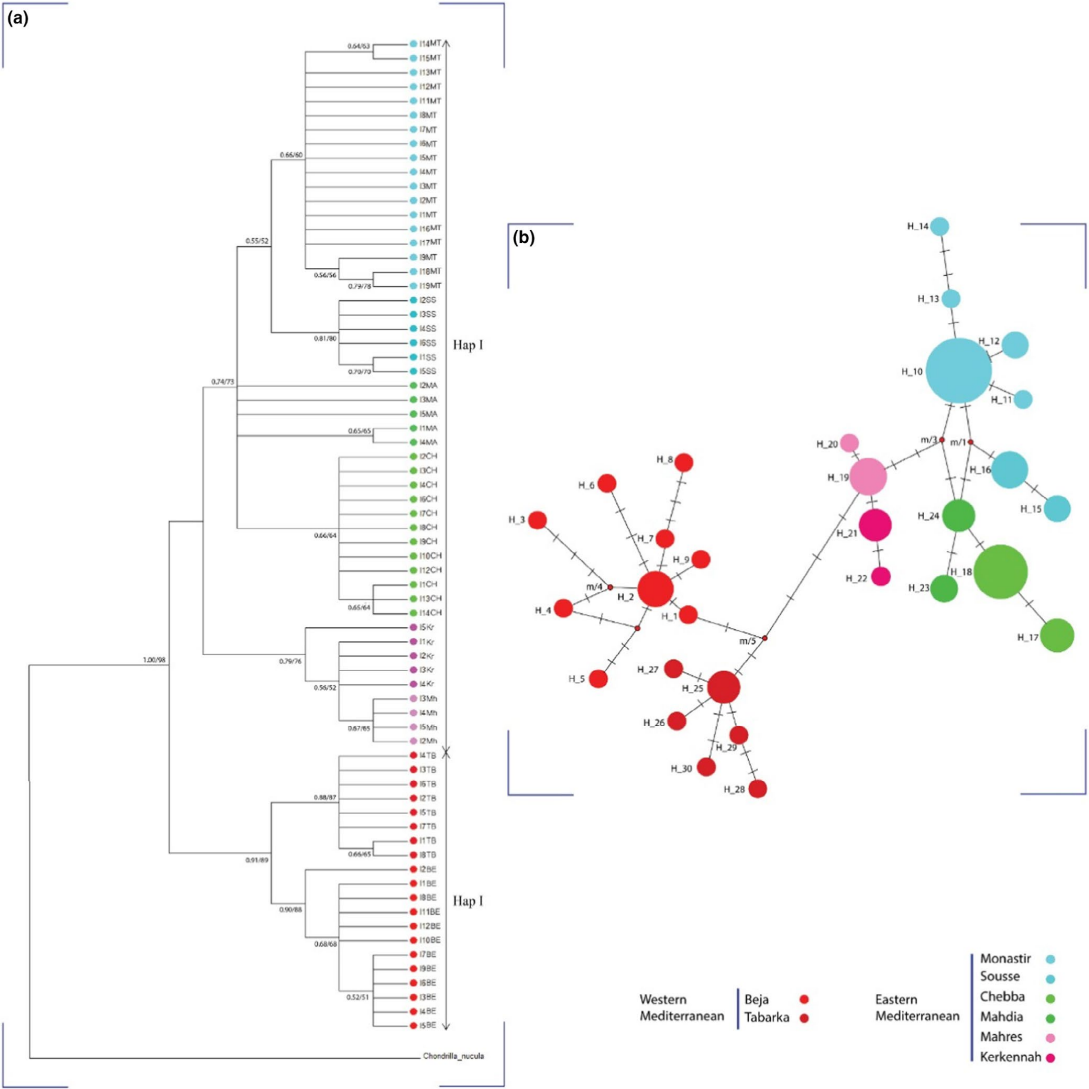
(a) Phylogenetic trees (Bayesian Inference/*Neighbor*‐*Joining*) and (b) Median‐joining network

The haplotype network, as well as the biogeographic trees, were built to discover genealogical relationships between *Chondrosia reniformis* haplotypes in Tunisia (Figure 4). Among the 70 sequences, 30 haplotypes were identified. Two clear haplogroups can be defined: Hap I grouping three groups Monastir/Sousse, Mahdia/Chebba, and Mahres/Kerkennah, the second Hap II corresponding to the Tabarka/Beja group. It is a clear distribution according to the geographic origin: Western Mediterranean and Eastern Mediterranean coasts. Haplotypes H2 (from Beja) and H10 (from Monastir) were the most branched haplotypes, which suggests that they are the ancestral ones.

To test the phylogenetic relationships between specimens, *Neighbor*‐*Joining* and Bayesian MCMC analyses were conducted. Both analysis built trees with strong values of bootstraps. All *C*. *reniformis* sequences were clustered into two major clades, strongly supported. Median‐joining network analysis showed the same subdivision of our populations (Figure 4): Western versus Eastern Mediterranean. It is noteworthy to mention that the group “Mahres/Kerkennah” diverges from eastern ones in a single sub‐clade.

The AMOVA test of Tunisian *C*. *reniformis* sponge revealed that 46.47% (ΦCT = 0.464, *p* < .05) of the genetic variation was detected between the four studied groups (Beja/Tabarka vs. Monastir/Sousse vs. Mahdia/Chebba vs. Mahres/Kerkennah). AMOVA results for the western Mediterranean and the eastern Mediterranean localities showed that more than 44% of the variation was between these two groups (ΦCT = 0.441, *p* < .05 (Table 4)); these haplogroups were suggested by the network and phylogeographic trees. “Mahres” and “Kerkennah” localities are parts from the Gulf of Gabes, this region of Mediterranean is well known to have extreme environmental conditions (Bejaoui et al., 2004; Ghannem et al., 2011), for that, we test the opportunity to have a specific genetic differentiation in this area. AMOVA results revealed that more than 73% of variation occurred between populations within this group.

**TABLE 4:** Molecular variance analysis (AMOVA) of *Chondrosia reniformis*, *p < .05

Source of variation	Fixation index	Sum of squares	Variance components	Percentage of variation
AMOVA groups: Beja/Tabarka vs. Monastir/Sousse vs. Mahdia/Chebba vs. Mahres/Kerkennah				
Among groups	*ΦSC* = 0.655	111.532	1.51315	46.46157*
Among populations within groups	*ΦST* = 0.815	36.817	1.14327	35.10428*
Within populations	*ΦCT* = 0.464	37.222	0.60036	18.43416*
Total		185.571	3.25677	100
AMOVA groups: Mahres/Kerkennah vs. Beja/Tabarka/Monastir/Sousse/Mahdia/Chebba				
Among groups	*ΦSC* = 0.660	17.604	0.24507	7.59179*
Among populations within groups	*ΦST* = 0.817	130.745	2.38263	73.81005*
Within populations	*ΦCT* = 0.452	37.222	0.60036	18.59817*
Total		185.571	3.94381	100
AMOVA groups: Western Mediterranean vs. Eastern Mediterranean				
Among groups	*ΦSC* = 0.727	67.441	1.73960	44.10959*
Among populations within groups	*ΦST* = 0.847	80.908	1.60385	40.66759*
Within populations	*ΦCT* = 0.441	37.222	0.60036	15.22282*
Total		185.571	3.94381	100

The AMOVA test of Tunisian C. reniformis sponge revealed that **46.47%** of the genetic variation was detected between the four studied groups. AMOVA results for the western Mediterranean and the eastern Mediterranean localities showed that more than **44%** of the variation was between these two groups. these haplogroups were suggested by the network and phylogeographic trees. “Mahres” and “Kerkennah” localities are parts from the Gulf of Gabes, this region of Mediterranean is well known to have extreme environmental conditions, for that, we test the opportunity to have a specific genetic differentiation in this area. AMOVA results revealed that more than **73%** of variation occurred between populations within this group.

The entire pairwise comparisons of groups based on *F*_ST_ and *N_m_* were significant (Table 5). The *F*_ST_ values between group pairs were considerable, indicating a high interpopulation divergence. Moreover, very low genetic values of gene flow (*N_m_*) were detected, indicating a remarkable differentiation among tested groups.

**TABLE 5:** Pairwise comparisons of genetic differentiation of *Chondrosia reniformis* estimated from haplotype frequency (*F*_ST_, above the diagonal) and gene flow (*N_m_*, below the diagonal)

	Monastir/Sousse	Mahdia/Chebba	Mahres/Kerkennah	Beja/Tabarka
Monastir/Sousse	0	0.605	0.703	0.66
Mahdia/Chebba	0.16	0	0.788	0.648
Mahres/Kerkennah	0.10	0.13	0	0.590
Beja/Tabarka	0.13	0.14	0.18	0

## 4 DISCUSSION

The cytochrome oxidase subunit I (COI) gene was amplified and analyzed for 70 Mediterranean *Chondrosia reniformis*. Polymorphism results revealed very low nucleotide diversity (***π***). These results were congruent with previous studies, which reported low sequence variation for mtDNA in several sponge species: *Crambe crambe*, *Astrosclera willeyana*, *Chondrilla nucula*, *Suberites diversicolor*, *Ianthella basta*, and *Xestospongia* spp (Andreakis et al., 2012; Becking et al., 2013; Duran et al., 2004; Duran & Rützler, 2006; Swierts et al., 2017; Wörheide, 2006). Moreover, a lower nucleotide diversity was reported for other Mediterranean marine animals using the same COI sequences (*π* = 0.0022, *π* = 0.0054, *π* = 0.0043, *π* = 0.0034 for *Penaeus melicertus*, *Palaemon elegans*, *Arbacia lixula*, and *Eriphia verrucosa*, respectively) (Deli et al., 2018; Deli, Kiel, & Schubart, 2019; Deli, Mohamed, et al., 2019; Zitari‐chatti et al., 2009).

Polymorphism results showed also that *C*. *reniformis* harbors high haplotype diversity (*H*_d_) throughout Tunisia coasts (*H*_d_ = 0.939). Compared with the previous data, this value was higher than that detected in other marine sponges (Andreakis et al., 2012; Becking et al., 2013; DeBiasse et al., 2010; Duran et al., 2004; Duran & Rützler, 2006). This value is also higher than that detected in other marines species in Tunisia: Green crab (Deli et al., 2015, 2017), Caramote prawn (Zitari‐chatti et al., 2009).

Diversity indices *H*_d_ and *π* were calculated to estimate the genetic architecture of populations and retrace possible historical events that may have acted on observed genetic diversity. It is generally accepted that small values of π suggest recently diverged populations due to founder effects or/and bottlenecks. Large values of π indicate deep genetic divergences between populations accumulated in isolation over long periods of time. According to Grant and Bowen (1998), the values of π vary from 0 to >0.1; the values close to 0 indicate the absence or presence of slight divergences between the sequences of the haplotypes, while values >0.01 suggest very important divergences between haplotypes and/or a secondary contact between differentiated populations. These authors introduced a framework of four categories to describe the histories of some marine organisms according to their indices of diversity *H*_d_ and *π*. The values of the diversity indices recorded in this study on *C*. *reniformis* from Tunisia (*H*_d_ = 0939, *π* = 0.0087) agree with the second category proposed by these authors (large value for *H*_d_ and small value for *π*; *H*_d_ > 0.5 and 0.5–0.8% < *π* ≤ 1%). Thus, we can consider that our sponge populations of *C*. *reniformis* may have undergone a bottleneck followed by rapid demographic expansion as mentioned by these authors for this category. This suggestion is strongly confirmed by the results of neutrality tests and “*mismatch distribution*.*”* However, the lower nucleotide diversity recorded could be derived from the synergy between the small sample size per population and the low polymorphism of mitochondrial region analyzed.

The important number of haplotypes between localities and the high genetic differentiation (*F*_ST_ ranged from 0.590 to 0.788) of the current *C*. *reniformis* populations, could be maintained by the limited gene flow. In this sense, very low genetic values of *N_m_* (0.10–0.18) were detected, indicating a remarkable genetic structuring of the tested groups. It is well known that gene flow in marine invertebrates is usually expected to be related to larval dispersal capacity or marine bio‐geographic barrier. Larval dispersal capacity strongly affects the geographic distribution and genetic differentiation of habitats (Deli et al., 2015; Kelly & Palumbi, 2010). However, decreased time that larvae spent in plankton is usually correlated with high differentiation among populations and vice versa (Avise, 1994; Scheltema, 1971). Several studies have reported that the dispersal ability of *C*. *reniformis* larvae is very low (Lazoski et al., 2001; Maldonado et al., 2021). The pelagic larval dispersal of *C*. *reniformis* is very short and lasts only a few days or even a few hours (Maldonado & Young, 1996; Uriz et al., 1998). Though, using allozyme marker, Lazoski et al. (2001) have revealed high genetic similarity between *Chondrosia* populations along Atlantic coasts of North and South America (Bermuda and Brazil). Indeed, contrary to what is expected, these authors found a fairly high gene flow (*N_m_* = 1.27) between populations (Lazoski et al., 2001). In these conditions, this unexpected find can be related to anthropogenic transport that had been reported for many marine invertebrate species (Holland, 2000).

Even though the genetic diversity of sequences was low, genetic differentiation was strong. Both haplotype Network and biogeographic trees analysis showed a structured distribution according to the geographic origin. The AMOVA analysis also confirmed the partition of genetic variation among populations. The current C. *reniformis* populations are subdivided into two major clades: Western and Eastern Mediterranean coasts. The same pattern of genetic differentiation has been previously observed in other Tunisian species, such as: the caramote prawn *Penaeus kerathurus* (Zitari‐Chatti et al., 2008), the brackish fish *Pomatoschistus tortonesei* (Mejri et al., 2009), the green crab *Carcinus aestuarii* (Deli et al., 2015), the banded Murex *Hexaplex trunculus* (Marzouk et al., 2016), and the black sea urchin *Arbacia lixula* (Deli et al., 2017). This pattern seems to be associated with the well‐known discontinuous biogeographic zone: the Siculo‐Tunisian Strait, which separates two water bodies circulating with different hydrological, physical, and chemical characteristics (Marzouk et al., 2016). The hydrodynamics was higher in the northern than in the southern Mediterranean coasts (Oueslati, 1993). According to Pinardi and Masetti (2000), the eastern Mediterranean Basin is characterized by very weak circulation.

The Siculo‐Tunisian Strait, from Cap‐Bon (Tunisia) to Mazara del Vallo (Sicily Island, southern Italy), has been inferred to be an oceanographic and biogeographic barrier between the two major Mediterranean sub‐basins (the western and eastern) (Bianchi & Morri, 2000).

On the other hand, Tunisian coasts are distinguished by a difference in temperature and salinity, the Eastern coasts being warmer and more saline than the Western ones (Serena, 2005).

**FIGURE 5:**
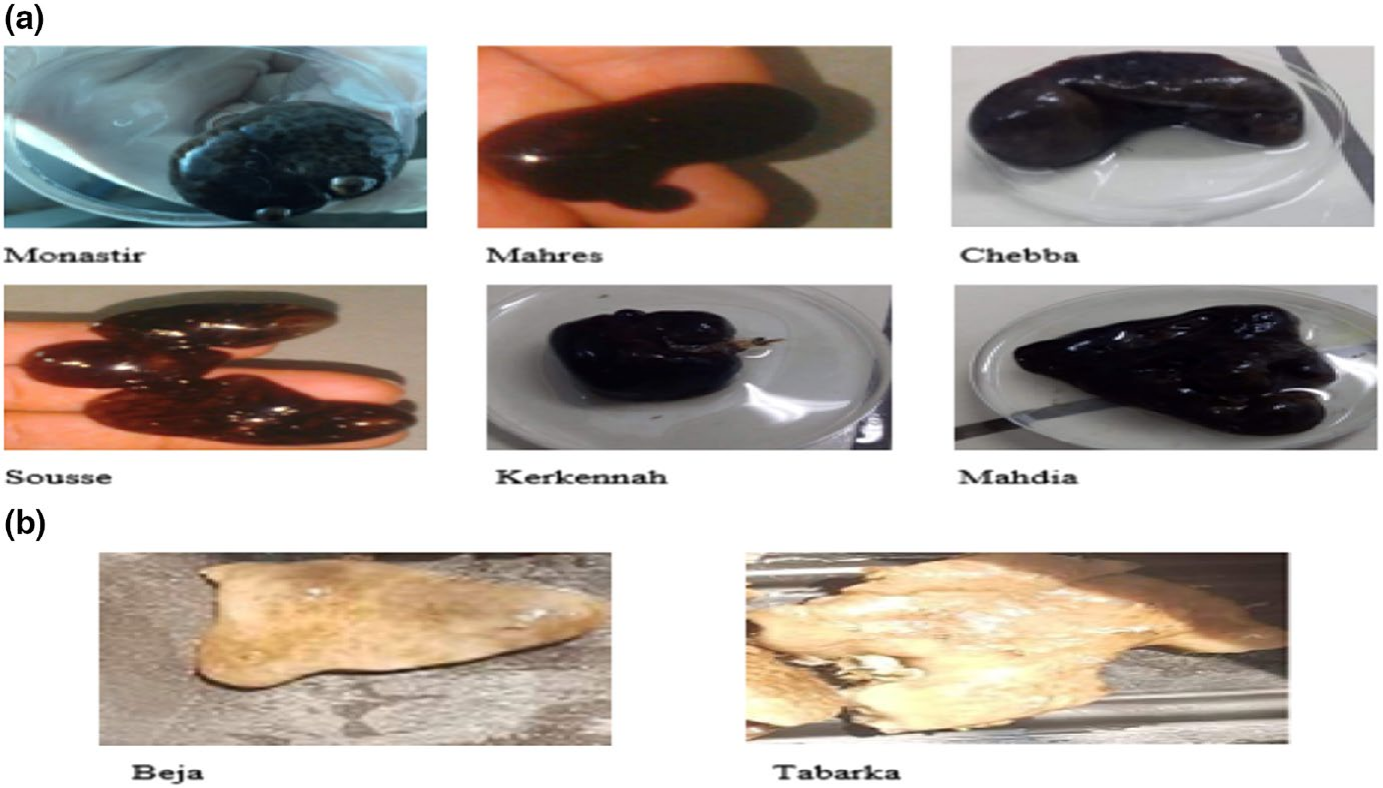
Photos of Western and Eastern *Chondrosia reniformis* specimens, (a) Eastern specimens, (b) Western specimens

In addition, the Tunisian coastline has different habitat textures varying from the muddy and sandy texture in the East to the rocky texture in the West. Due to the different geographical range of habitats, genetic differentiation between the western and the eastern Mediterranean populations of *C*. *reniformis* has been observed along the Tunisian coastline. This genetic differentiation was conformed to the apparent morphology of *C*. *reniformis* collected along this coastline. Thus, as shown in the photos (Figure 5), the Western *C*. *reniformis* specimens exhibit a light color and flattened shape, which contrasts with the Eastern specimens of dark color and lobed shape.

Additionally, it is noteworthy to mention that the “Mahres/Kerkennah” group diverged from Eastern ones in a single sub‐clade. This result was expected; indeed the region “Mahres / Kerkennah” belongs to the Gulf of Gabes, which represents a particular marine environment seriously influenced by phosphate industries. In fact, since the industrialization in 1970, the phosphogypsum discharge has been the main cause of the disequilibrium of this ecosystem of this important gulf. Currently, three regions, Sfax, Skhira, and Gabes generate phosphoric acid along the coasts and produce a large amount of phosphogypsum as a waste product (Bejaoui et al., 2004; Ghannem et al., 2011). The degradation of the ecosystem in these places has resulted in a degradation of water quality, a decrease in fish resources and a loss of marine biodiversity (Hamza‐Chaffai et al., 2003; Rabaoui et al., 2014; Salem et al., 2015).

The genetic divergence of populations of Mahres/Kerkennah group compared to those of the north and south of Siculo‐Tunisian strait in the sponge *C*. *reniformis* has not been observed for the other marine invertebrate species such as; the caramote prawn (Zitari‐Chatti et al., 2008, 2009), the green, and marbled Crab (Deli et al., 2015, 2017). For all these species analyzed, the populations of Gulf of Gabes did not show any differentiation. This can be attributed to the dominant sessile phase of the sponge life cycle and their filter lifestyle, which puts them directly in the face of selective pollution pressures.

Indeed pollution and climate change have created large dead zones in oceans; however, sponges are able to self‐organize and adapt more than any other species. They develop in the environments to which they have become accustomed over the millions of years of their evolution (Leys & Kahn, 2018; Müller & Müller, 2003). That versatility may be the key to their biodiversity even in polluted environments.

In summary, Tunisian *Chondrosia reniformis* evolution was affected by historical vicariance happening in Pleistocene glacial episodes. The variations in the sea's characteristics probably permitted the difference on either side of the Siculo‐Tunisian Strait. Sponge gene pools are under the control of physical and/or biological factors. The short dispersal of pelagic larvae of *C*. *reniformis* and marine biogeographic barrier created high differentiation among populations.

## CONFLICT OF INTEREST

The authors declare that there is no conflict of interest regarding the publication of this paper.

## AUTHOR CONTRIBUTION

**Moussa Maha:** Conceptualization (equal); Data curation (equal); Formal analysis (equal); Methodology (equal); Writing – original draft (equal); Writing – review & editing (equal). **Choulak Sarra:** Formal analysis (supporting); Methodology (supporting); Software (supporting); Supervision (equal); Writing – original draft (equal). **Rhouma‐Chatti Soumaya:** Supervision (equal); Validation (equal); Visualization (equal). **Chatti Noureddine:** Supervision (equal); Validation (equal). **Said Khaled:** Methodology (lead); Supervision (lead); Validation (lead); Visualization (lead); Writing – review & editing (lead).

## Data Availability

COI DNA sequences: Genbank accessions OL422911‐OL422980.
